# Nutrition Status of Children, Teenagers, and Adults From National Health and Nutrition Surveys in Mexico From 2006 to 2020

**DOI:** 10.3389/fnut.2021.777246

**Published:** 2021-11-25

**Authors:** Teresa Shamah-Levy, Lucia Cuevas-Nasu, Martín Romero-Martínez, Ignacio Méndez Gómez-Humaran, Marco Antonio Ávila-Arcos, Juan A. Rivera

**Affiliations:** ^1^Evaluation and Survey Center, Center for Evaluation and Survey Research, National Institute of Public Health, Cuernavaca, Mexico; ^2^Center for Mathematics Research, Unit Aguascalientes, Aguascalientes, Mexico; ^3^National Institute of Public Health, Cuernavaca, Mexico

**Keywords:** anthropometry, weight, height, body mass index, databases, national surveys, Mexico

## Abstract

**Background:** Population-level health and nutrition surveys provide critical anthropometric data used to monitor trends of the prevalence of under nutrition and overweight in children under 5 years old, and overweight and obesity in the population over 5 years of age.

**Objective:** Analyze the children malnutrition and overweight and obesity in children, teenagers and adults through the National Health and Nutrition Surveys information available from public databases.

**Materials and Methods:** Comparable anthropometric data was gathered by five Mexican National Health and Nutrition Surveys (in Spanish, ENSANUT). In pre-school-age children, under nutrition status was identified through underweight (Z-score below −2 in weight-for-age), stunting (chronic malnutrition) (Z-score below −2 for length/height-for-age), or wasting (Z-score below −2, for weight-for-length/height); overweight status was defined as a body mass index (BMI, kg/m^2^) for age over +2. For school-age children and adolescents, a Z-score BMI between +1 and +2 deviations was defined as overweight, and between +2 and +5.5 as obesity. In adults (≥20 years of age), overweight status was classified as a BMI between 25.0 and 29.9, and obesity as ≥30.

**Results:** The anthropometric data presented derives from the databases of five survey years of the Mexican National Health and Nutrition Survey: 2006, 2012, 2016, 2018, and 2020. They include a total of 210,915 subjects with complete anthropometric data (weight, length/height) distributed on five survey moments; subjects were categorized by age group: pre-school-age children (*n* = 25,968), school-age children (*n* = 42,255), adolescents (*n* = 39,275), and adults (*n* = 103,417). Prevalence of malnutrition by indicator was calculated: in pre-school-age children: low height- and weight-for-age, low weight-for-height, and overweight; and in school-age children, adolescents, and adults, the indicators calculated were overweight and obesity.

**Conclusions:** Results demonstrate the importance of maintaining systematic, reliable, and timely national anthropometric data in the population, in order to detect and track trends and to form the basis of nutrition-related public policy.

## Introduction

Reducing the global burden of malnutrition is a fundamental component of the United Nations Sustainable Development Goals, which include monitoring indicators of under nutrition, overweight, and obesity.

Mexico has a history of over three decades conducting health and nutrition surveys as part of a nationwide monitoring system. The National Health Surveys System in Mexico includes a series of multi-thematic surveys on health and nutrition topics; surveys are probabilistic and nationally representative. The results of these surveys have been key for evaluating the performance of the Mexican health system, providing precise, detailed, and representative information about the status of health and nutrition of the population and for planning health and nutrition policies.

The first national health and nutrition surveys were conducted by the Secretariat of Health (SoH) or the National Public Health Institute (INSP) and the operatives of the Health surveys were different from those of the nutrition surveys which only included mothers and children (1, 2).

In 2006, the nutrition and health surveys were combined into a single Mexican National Health and Nutrition Survey (in Spanish, ENSANUT), which has since been applied four times, all of them conducted by INSP using similar methods and sampling procedures in order to allow comparison: 2006 (3), 2012 (4), 2016 at “*Medio Camino (MC)*” (halfway point) (5), and 2018–2019 (6). The surveys, with the exception with the one collected in 2016, were conducted every 6 years, at the end of the Federal Administrations.

Beginning in 2020, with the support of the Health Secretariat, ENSANUT is now a continuous survey, with data collection every year, so that within a 5-year time window a sample is created which is representative at the national level, urban/rural level, and most importantly, by state level. Notably, the 2020 ENSANUT obtained data aimed at updating the general landscape on the frequency, distribution, and trends of health and nutrition status indicators and their determinants, but in addition, it includes a specific COVID-19 component which measured SARS-CoV-2 antibody seroprevalence in a population subsample, which allows an estimation of the proportion of the population with possible exposure to the coronavirus infection. In addition, the information collected provides a closer look at experiences and changes in behavior, food security, diet, physical activity, and healthcare seeking behavior of the Mexican population stemming from the COVID19 pandemic and the measures adopted to contain it, including the way in which individuals have dealt with the confinement period and to what extent they have adopted mitigation measures.

In light of the poverty and inequality persistent in Mexico, one critical factor driving the creation of the Mexican nutrition surveys was the need to monitor the nutritional status of children under 5 years old. For three decades, these surveys have documented national trends in under nutrition for children under five—recognized since the 1980's as an immense public health challenge in the country—offering data disaggregated by age, sex, geographical region, and socioeconomic level. On the other hand, an epidemic of overweight and obesity in all age groups in Mexico has also been documented in the last three decades, in addition to other serious public health problems in the population (7–9). The ENSANUT 2021 is currently underway and will generate information on progress made and challenges which remain, as well as supporting the identification of health and nutrition priorities for the coming years and necessary strategies given the ongoing COVID-19 epidemic in Mexico.

The goal of this study was to review the anthropometric data available through national databases in Mexico, which are key to tracking nutritional status indicators including under nutrition, overweight and obesity in the population. These indicators may be used to inform and craft nutrition-specific public health policy based on transparent, reliable, updated, and timely information in Mexico.

## Materials and Methods

The ENSANUT is a probabilistic household-level survey, which assigns to each Mexican household a known value >0 as probability of selection. The ENSANUT is a stratified cluster survey, with strata defined by population size of the locality of interest: rural (1–2,499 inhabitants), urban (2,500–99,999 inhabitants), or metropolitan (≥100,000 inhabitants). Primary Sampling Units (PSU) of ENSANUT were geographical areas defined by INEGI (National Institute of Geography and Statistics): ENSANUT 2018 used clusters of the Master Sampling Frame of INEGI for surveys, and the rest of ENSANUT used the Basic Geographical Areas (AGEB). ENSANUT 2016 and ENSANUT 2020 were designed to make inferences on regions: ENSANUT 2020 (nine regions) and ENSANUT 2016 (four regions); and ENSANUT 2006, 2012, and 2018 were designed to make inferences on the States of México (32). As an example of parameter used to estimate the sample size, we present the sample size of ENSANUT 2018. For the planning of ENSANUT 2018, a deff = 2.0 was used, a value that was estimated from the experience in surveys carried out by INSP and INEGI. The sample size by state was calculated with the formula: $n=Z^{2}\frac{p\left(1-p\right)}{\delta^{2}RK}$ Deff.

where *n* = sample size in households, p = proportion to estimate, Z = Quantile 97.5% of a unit normal distribution (Z = 1.96), δ = is the semi-amplitude of the confidence interval, R = expected response rate (85% in dwellings, 85% in adults and adolescents, 88% in schoolchildren, and 90% in pre-school), K = number of individuals expected to obtain from the interest group (pre-school K = 0.24, schoolchildren K = 0.26, adolescents K = 0.48, adults K = 0.99). Based on the assumed parameters, a sample size of 1,580 homes per state was chosen. The sample size will allow estimating prevalences of 10% with the following semi-amplitudes: 2.54% in adults, 3.57% in adolescents, 5.14% in schoolchildren, and 4.91% in preschool.

In general, the ENSANUT allocates sample sizes by State, seeking to ensure that all households have a similar probability of selection and selects clusters with probabilities proportional to their population.

Further details of sampling procedures and data results are discussed in previous articles and are publicly available through the website www.ensanut.insp.mx, which allows users to download data and survey instruments.

The ENSANUT disaggregates the population by four age groups: pre-school-age children (0–4 years), school-age children (5–9 years), adolescents (10–19 years), and adults (≥20 years). Selection procedures differ by age group. One adolescent is selected to represent the adolescents of a household. For pre-school-age and school-age children, the selection procedure has evolved over time; in 2006 one child between zero and 9 years of age was selected for each household; ENSANUT 2012 and 2016 selected both one pre-school-age and one school-age child per household, and since ENSANUT 2018 all pre-school-age children are included and only one school-age child is selected. From ENSANUT 2006 to 2018, only one adult per household was selected; however, in the 2020 survey one adult might be selected from each of the following age groups: 20–34, 35–49, and 50 years and above. Each person selected from a household was interviewed and his or her anthropometric measurements were taken by a group of trained specialists following a standardized protocol which guaranteed comparability between measurements of different surveys.

Anthropometric data was standardized in body weight and height measurements according to international standards (10, 11). In the case of the pre-school-age population, weight-for-height, and weight- and height-for-age indexes were constructed, which were then converted to Z-scores according to World Health Organization (WHO) standards (12). Participants were classified as under nutrition status was identified through underweight (Z-score below −2 in weight-for-age), stunting (chronic malnutrition) (Z-score below −2 for length/height-for-age), or wasting (Z-score below −2, for weight-for-length/height); overweight status was defined as a body mass index (BMI, kg/m^2^) for age over +2. In the school-age and adolescent groups, BMI (kg/m^2^) was calculated by age and sex and categorized according to WHO guidelines (13) as overweight when BMI Z-score was between +1 and +2, and obese when Z-score was between +2 and +5.5. For adults, WHO classification standards were also applied: overweight was considered as BMI of 25.0–29.9, and obesity as BMI ≥ 30 (14).

### Ethics Statement

ENSANUT protocols were approved by the committees of Ethics, Biosecurity and Research of the National Institute of Public Health of Mexico, and all participants signed an informed consent form. The data contained in the databases complies with the guidelines established by the Ethics Committee and national laws regarding confidentiality and transparency. Under no circumstances data that could reveal the identity of study participants is disclosed.

## Results

### Relevance of Public Health Databases Use

Since 2006, anthropometric data has been one of the main outputs of the Mexican National Health and Nutrition Surveys, gathered across all survey years and for all age groups (pre-school-age to seniors) (Table 1). Related results have been specifically disaggregated, analyzed and reported within public reports, and have emerged as a topic of significant interest to the public health community, decision makers, and the general population. The corresponding datasets have provided information on the nutrition status of the population across survey years, and perhaps most importantly, through a transversal comparable design, have also revealed trends which have alerted about the increasing trends of overweight and obesity, which can be characterized as epidemics; at the same time it has informed about the decline of stunting in general and its persistence in some marginalized subgroups.

**Table 1:** Anthropometric information available in databases in Mexico, by age group and survey year.

**SURVEY**	**Format(s)**	**Description**	**Measurement**	**Age groups**										**Representativity**	**Extra variables/info**
				**Preschool 0–4**		**School 5–11**		**Teenagers 12–19**		**Adults #x02265;20**		**Total**			
				***n***	***N***	***n***	***N***	***n***	***N***	***n***	***N***	***n***	***N***		
2006	SPSS (^*^.sav)	One single SPSS file including all the population groups for the survey	Weight	7,725	9,441,669	15,165	15,885,880	14,794	18,962,586	33,785	59,011,558	71,469	103,301,693	Urban/Rural localities inside the four regions of the country	Includes waist circumference for adult population
			Height	7,725	9,441,669	15,165	15,885,880	14,794	18,962,586	33,785	59,011,558	71,469	103,301,693		
2012	SPSS (^*^.sav), STATA (^*^.dta), CSV	One single SPSS file including all the population groups for the survey, additional file including survey dates and a processed version of the dataset that include variables with score-z and BMI computations	Weight	10,887	10,966,681	16,484	16,565,012	14,214	18,308,611	38,267	69,245,519	79,733	115,085,824	Urban/ Rural inside any of the 32 states that conform the country	Includes blood pressure, calf, waist and hip circumference, toe to knee distance, and demi-span
			Height	10,853	10,966,681	16,476	16,565,012	14,207	18,308,611	38,218	69,245,519	79,733	115,085,824		
2016	SPSS (^*^.sav), STATA (^*^.dta), CSV	This data corresponds to the mid-point 2016 survey, one file includes all the age-groups	Weight	2,028	11,104,791	2,391	11,602,871	3,431	23,045,978	8,435	70,888,336	16,285	116,641,975	Urban/Rural localities inside the four regions of the country	Includes blood pressure, calf, arm, waist and hip circumference, toe to knee distance, and demi-span
			Height	2,020	11,031,753	2,391	11,602,871	3,430	22,997,366	8,413	70,760,153	16,254	116,392,142		
2018	CSV	One single CSV file including all the population groups for the survey	Weight	3,776	9,736,969	6,268	11,004,874	5,672	22,904,289	13,053	61,793,694	28,769	105,439,826	Urban/Rural inside some selected states of the country ^*^	Includes blood pressure, calf, arm, waist and hip circumference, toe to knee distance, and demi-span, this is the only survey conducted along with the National Statistics and Informatics Institute (INEGI)
			Height	3,998	9,737,540	6,268	11,004,874	5,672	22,904,289	13,012	61,587,914	28,950	105,234,617		
2020	SPSS (.sav), STATA (.dta), CSV	One single file including all the population groups for the survey	Weight	1,594	10,097,632	1,955	15,132,188	1,172	17,545,092	9,989	83,869,561	14,710	126,644,473	Urban/ Rural inside any of the 32 states that conform the country	Includes blood pressure and a post-sampling generated variable to expand to the 2020 census reported population. This is the first edition from the “continuous” mode of the survey and was conducted during COVID-19 pandemic
			Height	1,594	10,097,632	1,955	15,132,188	1,172	17,545,092	9,989	83,869,561	14,710	126,644,473		

*Source: ENSANUT 2006, 2012, 2016, 2018–19, and 2020*.

In regards to data quality, in addition to the standardization process required by personnel responsible of the anthropometric measurements, data was recorded using a software which required that at least two duplicate measurements be taken, allowing data collection to proceed only if the measurements differed by no more than 200 g for weight in children and 400 g in adults, and 4 mm for height for both age groups; this was intended to minimize errors related to recording, dictation, or measurement. Finally, the software also addressed sampling and data-integrity issues, allowing data collection only from those individuals previously selected in the sampling process and guaranteeing through unique identification codes that every anthropometric measurement link to its counterparts from the other survey sections (e.g., household characteristics, health indicators, or feeding practices).

Since all data obtained in the surveys by the National Institute of Public Health received public funding, datasets from all survey years may be downloaded freely from the ENSANUT website (https://ensanut.insp.mx/). For each survey year, a link reading “*Descargar bases*” (Download datasets) leads the user to data gathered, ordered by survey topic and population. Popular data processing systems such as SPSS, STATA, or the non-proprietary CSV format are the ideal options for dataset download, or alternatively, the paper version of the survey instrument in PDF and a catalog in Excel may be downloaded from the same website for each survey topic. SPSS and STATA files, in addition to the provided reference index, are labeled by variable and value, and missing values are declared. All data files include, in addition to information about the section, the key variables used at household and individual level in order to enable file merges with other ENSANUT datasets on topics such as disease, household characteristics, blood samples, healthcare service usage, and others. Another important feature is that the public datasets include geographic variables which allow all surveys to be identified by municipality (county); notably, although the sampling methodology identifies both households and street blocks, due to confidentiality laws the only data available to the public is at county level. Finally, all sampling variables are included in the datasets: design strata, PSU, and weights which allow the calculation of confidence intervals for and population parameters. Datasets also include auxiliary variables commonly used in anthropometric calculations or to filter observations, such as gender, age, birth, survey date, pregnancy, disability, and clothing characteristics in the case of pre-school-age children.

### Database Structure Description

In the Table 2 the complete structure of one of the anthropometric tables (ENSANUT 2018) is presented as an example. The included data for every variable is: name, data type, width, and an English-translated version of the question, it is important to mention that this last field (in Spanish) corresponds exactly to the question in the PDF of the questionnaire as well as the data entry application. In the documentation, the range of valid and missing values are also included. In those variables that applies a translated list of value labels (codes) is also provided.

**Table 2:** The complete structure of one of the anthropometric tables (ENSANUT 2018).

**TABLE NAME: Antropometr**í**a**							
**ID**	**Variable name**	**Question (English)**	**Type**	**Length**	**Valid codes**	**Value labels**	
						**Numeric**	**Label**
**1**	**UPM**	**Primary sampling unit**	**C**	**5**	**00001…03938**		
**2**	**VIV_SEL**	**Selected residential house**	**C**	**2**	**01…25**		
**3**	**HOGAR**	**Household number**	**C**	**1**	**1…4**		
**4**	**NUMREN**	**Family member id**	**C**	**2**	**01…19**		
5	PESO1_1	Now I will weight (Name) Kg/g first Measurement	C	7	002.500…222.220, 222.222,b	222.222	Not weighted
6	PESO1_2	Now I will weight (Name) Kg/g second Measurement	C	7	002.500… 222.220,b		
7	P2	Clothes	C	1	1.0.0.4,9,b	1	Light
						2	Thick
						3	Without clothes
						4	Not weighted
						9	Don't know
8	P3	Weight result	C	1	1.0.0.3,9,b	1	Without problem
						2	Physical problem
						3	Did not cooperate
						9	Don't know
9	TALLA4_1	Now I will measure the height of (name) cm first measurement	C	5	045.5…196.2,222.2,b	222.2	Not measured
10	TALLA4_2	Now I will measure the height of (name) cm first measurement	C	5	045.5…196.4,b		
11	P5	Height result	C	1	1.0.0.3,9,b	1	Without problem
						2	Physical problem
						3	Did not cooperated
						9	Don't know
12	P6	Are you …	C	1	1.0.0.4, 9,b	1	Pregnant?
						2	Breastfeeding?
						3	Pregnant and breastfeeding?
						4	None of the previous
						9	Don't know
13	P7_1	How many months of pregnancy?	C	2	01…09, 99,b	99	Not specified
14	CIRCUNFERENCIA8_1	Now I will measure the waist of (name) first measurement	C	5	011.4…187.8,222.2,b	222.2	Not measured
15	CIRCUNFERENCIA8_2	Now I will measure the waist of (name) second measurement	C	5	011.3…187.1,b		
16	P9	Result of waist measurement	C	1	1.0.0.3,9,b	1	Without problem
						2	Physical problem
						3	Did not cooperate
						9	Don't know
17	P10	Did (name) lose weight recently (in the last 3 months)?	C	1	1.0.0.3, 8, 9,b	1	No weight lost
						2	Lost between 1 and 3 kg
						3	Lost more than 3 kg
						8	Not responding
						9	Don't Know
18	P11	(Name) suffered any limb amputation?	C	1	1,3.0.0.6,b	1	Yes, upper limbs and is able to walk
						2	Yes, upper limbs and is unable to walks
						3	Yes, lower limbs
						4	No, and he/she can stand up
						5	No and he/she can only be seated
						6	No, and is bedridden
19–25	These are the same variables as P5–P11 but only for individuals over 60 years old, that can stand.						
26	TALLAPIE17_1	Now I will measure the distance between the heel and knee of (name), cm	C	5	021.0…158.0,222.2,b	222.2	Not measured
27	P18	Heel-Knee measurement result	C	1	1.0.0.3,b	1	Without problem
						2	Physical problem
						3	Did not cooperate
28	CIRCPANTORRILLA19_1	Now I will measure the thigh circumference of (Name) cm	C	5	016.6…89.9,222.2,b	222.2	Not measured
29	P20	Thigh circumference measurement result	C	1	1.0.0.3,b	1	Without problem
						2	Physical problem
						3	Did not cooperate
30–32	These are the same variables as P14–P16 but only for individuals over 60 years old, that can stand.						
33	HEMIENVERGADURA23_1	Now I will measure the distance from the chest to the medium fingertip of (name) cm	C	5	070.0…095.1,222.2,b	222.2	Not measured
34	P24	Half arm-span measurement result	C	1	1.0.0.3,b	1	Without problem
						2	Physical problem
						3	Did not cooperate
35	MEDIABRAZO25_1	Now I will measure the mid-arm circumference of (name) cm	C	5	022.3…037.8,222.2,b	222.2	Not measured
36	P26	Mid-arm circumference measurement result	C	1	1.0.0.3,b	1	Without problem
						2	Physical problem
						3	Did not cooperate
37	P27_1_1	Now I will measure the blood pressure of (name) first measurement, systolic	C	3	059…255,b		
38	P27_1_2	Now I will measure the blood pressure of (name) second measurement, systolic	C	3	040…193,222,b	222	Not measured
39	P27_2_1	Now I will measure the blood pressure of (name) first measurement, diastolic	C	3	056…256,b		
40	P27_2_2	Now I will measure the blood pressure of (name) second measurement, diastolic	C	3	000…191,b		
41	P28	Blood pressure, time of measurement (hours: min)	C	5	01:00…23:59		
42	P29	Arm used to measure blood pressure	C	1	1.0.0.3,b	1	Left arm
						2	Right arm
						3	Not measured
43	P30	Blood pressure measurement result	C	1	1.0.0.4,b	1	Without problem
						2	Physical problem
						3	Did not cooperate
						4	Denial
**System Variables**							
44	EDAD	Age in years	C	3	000…111	000	Under 1 year old
45	EDAD_MESES	Age in months	C	4	0…1337		
46	SEXO	Gender	C	1	1,2	1	Male
						2	Female
47	ENT	State code	C	2	01.0.0.32		
48	DOMINIO	Locality domain	C	1	1,2	1	Urban
						2	Rural
49	ALTITUD	Altitude in meters over sea level	C	4	0…3241		
50	REGION	Region of the country	C	1	1…4	1	North
						2	Center
						3	Mexico city
						4	South
51	EST_DIS	Design strata	C	3	001…301		
52	UPM_DIS	Design psu	C	5	00001…03938		
53	ESTRATO	Sociodemographic strata	C	1	1…4		
54	F_ANTROP	Original weight	C	6	141…132198		
55	F_ANTROP_INSP	Calibrated weight	C	6	0…132198		
56	GPO_INSP	Age group	C	1	0…4	1	Pre-school children
						2	School children
						3	Teenagers
						4	Adults
57	DIAS	Age in days	C	5	9…38542		

The ENSANUT data files always begin with the key variables (ID: 1–4) that uniquely identify every observation (households or individuals), this fields allow all the merge operations with other survey sections, following (ID: 5–16) the anthropometric variables can be found, including the repeated measurements and additional control variables like the clothes type, measurements result for every indicator, and pregnancy. The variables ID: 17 and 18 are used to register weight loss and limb amputations. The variables corresponding to 19–25 IDs are the same as 5–11 but adapted to individuals of 60 and more years old. The variables (ID: 26–36) are gathered to estimate the actual height using proximal indicators like heel-knee height or mid arm span for people that are unable to stand, here, some body composition variables like thigh or mid-arm circumferences are located. After this, the table contains the blood pressure variables (ID: 37–43) and finally, a group of variables entitled “System variables” (ID: 44–57) that include geographic variables (state code, altitude), analysis variables often used in the crosstabs creation of the surveys (gender, age groups, Country region, type of locality, etc.); in this section are also included, all the sampling related variables (strata, PSU, weights) used to estimate variances.

### Database Use

**Figure 1:**
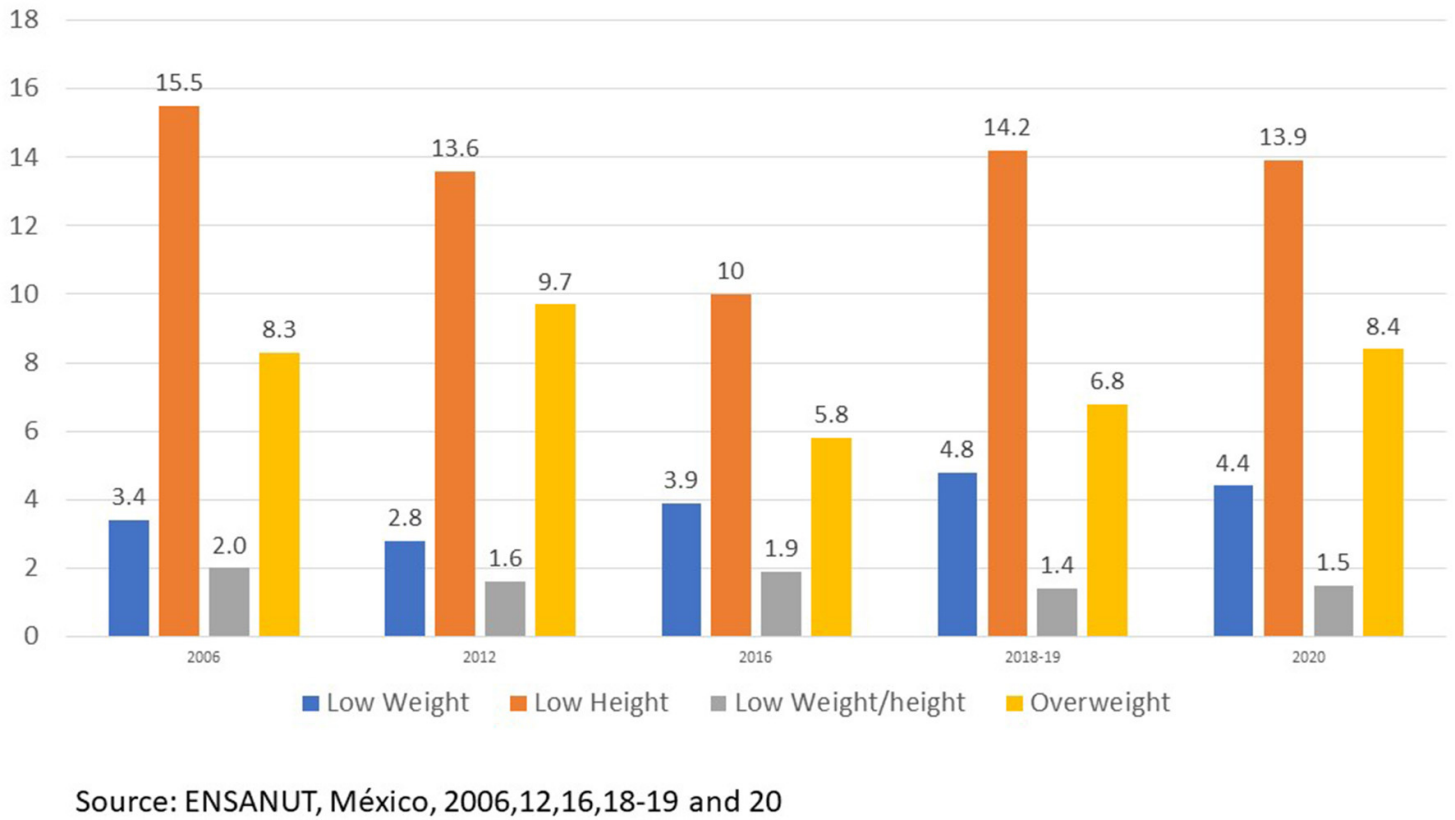
Prevalences of nutrition status in children <5 years age by year survey in Mexico. *Source: ENSANUT 2006, 2012, 2016, 2018–19, and 2020*.

Prevalences of malnutrition: low weight, stunting, and wasting in children under 5 years old and overweight and obesity for all the selected population were obtained. Figure 1 shows that the stunting prevalence in 2006 was 15.5% then decreased to 10% in 2016 and finally increased up to 13.9% in 2020. This, constitutes one of the main public health problems in population under 5 years old in Mexico (15, 16).

**Figure 2:**
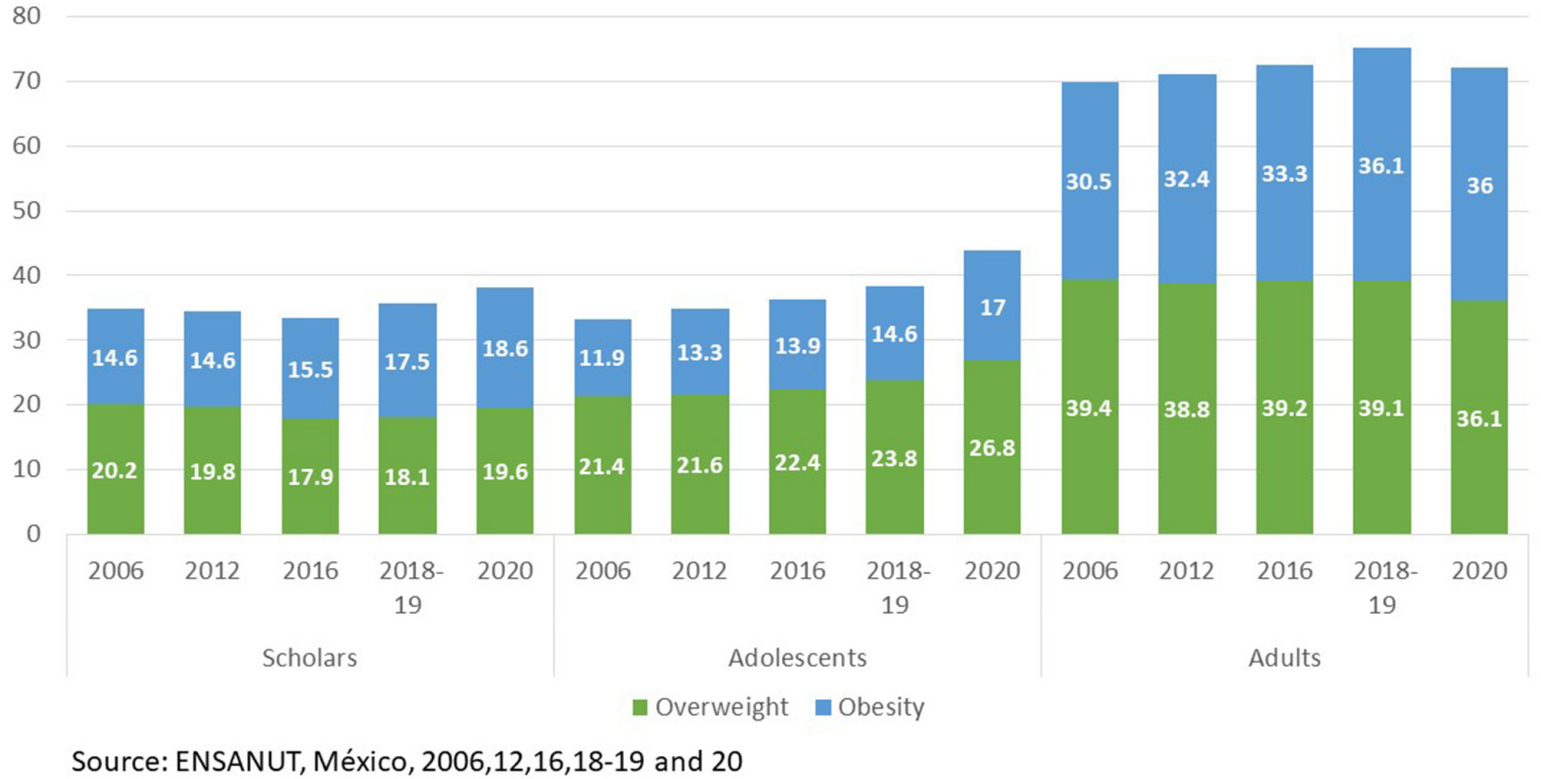
Prevalences of overweight and obesity by age group and year survey in Mexico. *Source: ENSANUT 2006, 2012, 2016, 2018–19, and 2020*.

Figure 2 shows the trends of overweight and obesity prevalences among the population groups. For school age and teenagers excessive weight is above 30% whereas in adult population this cipher exceeds 70%.

Back in 2006, with the results obtained through ENSANUT variables analysis. Mexico was declared at national alert due to the weight excess in the population. This triggered the development of National public policies aimed to immediately address this problem (7, 8).

## Discussion

This study presents the anthropometric data available at a national level for inhabitants of Mexico. ENSANUT can explore information by characteristics such as area of residence (urban or rural), geographical region, state, age group, and sex. The findings highlight the availability of comparable, consistent, transparent, and high-quality nutrition data which can be used to support local-level analyses.

High-quality and up-to-date anthropometric data serves as the basis for the evaluation of combined nutritional status indexes of weight, age, and height, and helps to make the difference between high-quality surveys and those with low-quality data. In the context of surveys like the ENSANUT which are applied periodically, the validity, and robustness of the data collected allows estimation of the changes over time of the prevalence of nutritional status indicators of the Mexican population (see Supplemental Material) (17).

Previous studies have demonstrated that investments toward improving the quality of data collection procedures improves the quality of anthropometric data (18). However, variations in survey quality are also related to contextual factors which present challenges to successful implementation: for example, conflict, political instability, or geographically isolated populations.

Among the strengths of this study is that explore data from the different ENSANUTS; it is worth noting that to our knowledge, Mexico is one of the first countries in Latin America to make this data freely available to the public. The methodological alignment between the distinct ENSANUT survey all analysis and comparison across five time points, which may be further explored through characteristics such as area type (urban or rural), or ecological variables from robust and reliable sources. Another strength of this study is the probabilistic design, which allows estimations from a sample representative at the national, regional and area (urban or rural) level, and comparisons among previous national health and nutrition surveys with similar methodologies. This contributes to the overarching goal of track trends, inform public policy makers, and contribute to the reformulation of actions seeking to prevent and treat malnutrition and its outcomes among different age groups in Mexico.

Limitations of our study include the sample size of anthropometric data on child malnutrition in the ENSANUT-MC 2016, since although this “halfway point” survey shared the same sampling design as other survey years, its focus was to identify overweight and obesity. The same should be noted for survey year 2020, considering that sample size for states should be completed in 2024.

## Conclusion

In conclusion, the periodic collection and availability of population-level anthropometric data is a key component of a system which monitors and evaluates national nutrition trends and allows comparison at the local, national, regional, and global level. The availability of this data implies a nationwide effort which serves to: provide critical data to determine the magnitude, distribution, and trends in population nutrition; maintain systematic, reliable, and timely information based on multiple points of analysis; and inform the formulation and evaluation of national public policy around nutrition and associated factors.

## Data Availability Statement

The original contributions presented in the study are included in the article/Supplementary Material, further inquiries can be directed to the corresponding author/s.

## Ethics Statement

The studies involving human participants were reviewed and approved by the Research Ethics Committee of the National Public Health Institute. Written informed consent to participate in this study was provided by the participants' legal guardian/next of kin.

## Author Contributions

TS-L, JR, and LC-N: conceptualization and investigation. MR-M, IG-H, and MÁ-A: methodology. MÁ-A: analysis. TS-L: writing—original draft. MR-M, LC-N, and JR: writing—review and editing. All authors contributed to the development of this manuscript and read and approved the final version.

## Funding

All the National Health and Nutrition Surveys were funded by the Ministry of Health of Mexico, and in 2018 also by the Geographic and Statistic Institute of Mexico (INEGI).

## Conflict of Interest

The authors declare that the research was conducted in the absence of any commercial or financial relationships that could be construed as a potential conflict of interest.

## Publisher's Note

All claims expressed in this article are solely those of the authors and do not necessarily represent those of their affiliated organizations, or those of the publisher, the editors and the reviewers. Any product that may be evaluated in this article, or claim that may be made by its manufacturer, is not guaranteed or endorsed by the publisher.

## Supplementary Material

The supplemental materials include anthropometric information available by survey year and age group, converted to prevalences, and trends over time (2006–2020).

Supplementary Table 1 Nutritional status of the population of children under 5 years of age, as evaluated by weight-for-height/length, height/length-for-age, weight-for-age, and presence of obesity.

Supplementary Table 2 Prevalence of overweight and obesity in school-age children (5–11 years).

Supplementary Table 3 Prevalence of overweight and obesity in adolescents (12–19 years).

Supplementary Table 4 Prevalence of overweight and obesity in adults (≥20 years).
