# Supplementary material for: Nutrition Status of Children, Teenagers, and Adults From National Health and Nutrition Surveys in Mexico From 2006 to 2020
Source: Front Nutr. 2021 Nov 25;8:777246. doi: 10.3389/fnut.2021.777246 (PMC8656215; doi:10.3389/fnut.2021.777246)
Supplement: Supplementary file 2 [file Data_Sheet_1.docx]

Appendix

Validity of ENSANUT 2018

1. Summary

ENSANUT 2018 is a probabilistic survey designed to achieve two goals: a) To describe the health and nutritional status of the 32 states of México , and b) to estimate national trends of the main chronic diseases (diabetes, hypertension and obesity). Probabilistic surveys are exercises of statistical inference; this is, they try to make inferences from a sample to the population. Statistical inferences from a survey can be expressed through confidence intervals, and validity of confidence intervals can be supported in two ways :

- Theoretically, if a survey is probabilistic and measurements have no error, the intervals with 95%-confidence for a parameter (θ) should contain the parameter in 95% of the times that a confidence interval is obtained. Therefore, we describe the sampling procedure of ENSANUT 2018 to show that it is a probabilistic survey and to support the validity of the confidence intervals when measurements have no error.
- Practically, we verify that estimations of ENSANUT 2018 for parameters that change slowly on time are similar to estimations of other surveys. In particular, we show that a measurements of glucose in a sub-sample of ENSANUT 2018 provides estimates of the prevalence of diabetes that are congenial to results of ENSANUT 2020 .

1. Sampling design

The usual way to make a probabilistic survey is to: construct a list of subjects (sampling frame), allocate probabilities of selection, and select a sample.

2.1 Sampling frame and selection of Primary sampling units (PSU)

The National Institute of Geography and Statistics (INEGI) defined, in the year 2012, a set of geographic areas as a primary units sampling (PSU). PSU were defined from the cartographic and demographic information of the Population and Housing Census 2010; the PSU´s were classified by three criteria: state, size of the locality and socio-demographic conditions of the households. Mexico has 32 States, and four strata were formed through the statistical summary of 34 indicators that describe the physical characteristics and equipment of the households of the PSU, as well as characteristics sociodemographic of the inhabitants of the households. On the other hand, three strata of localities were formed according to its number of inhabitants: rural (localities with less 2,500 inhabitants), high urban (cities with 100,000 or more inhabitants) and the urban complement (localities with more than 2,499 and less than 100,000 inhabitants). Once the PSU´s and strata were built, the PSUs of the Ensanut 2018 were selected in two stages: first, INEGI selected a master sample of PSU´s with probability proportional to its number of households in the year 2012; then, INEGI selected a subsample of PSU with equal probability within each stratum in 2018. The number of PSU to select in the strata and the number of households to select in the PSU were set to look for a self-weighted design. Finally, in each PSU, households were selected with equal probability; on average, five households were selected in each PSU of the upper urban stratum, and 20 households were selected in each PSUs of the strata : rural and urban complement.

Selection of individuals in households

A household questionnaire was applied to each household, which listed and stratified all the inhabitants into six age groups. In the second stage, a sampling fraction (Table 1) was applied in each age group. Additionally, ENSANUT 2018 selected a sample of health service users whom received outpatient medical in the last two weeks.

Table 1. Sampling fraction for individuals in the household

| Group | Fraction of selection |
| --- | --- |
| Preschool Children from 0 to 4 years old | One per household |
| School Children 5 to 9 years old | One per household |
| Adolescents 10-19 years old | One per household |
| Adult 20-34 years old | One per household |
| Adult 35-49 years old | One per household |
| 50 years old and over | One per household |
| Health service user | Up to 2 in 40% of the households |

Therefore, ENSANUT 2018 selected individuals with a known probability, which was used to calculate the sampling weights. Sampling weights of ENSANUT 2018 were calculated on the basis of : a) probabilities of selection, b) response rates, and c) government estimations of the total number of individuals of México. We expect that ENSANUT 2018 produces unbiased estimators because weights were derived from probabilities of selection, and several ENSANUT COVID 19 estimators resulted congenial to estimators of previous surveys, as is exemplified next.

Results

The sample was distributed into 32 states, 798 municipalities and 6,268 primary sampling units. Additionally, Ensanut 2018 obtained response rates greater than planned, 50,654 homes were visited and response was obtained in 44,069 dwellings (87%) for all their households (44,612).

1. Survey validation

We present only two items for validation: the age pyramid and the prevalence of diabetes.

1. Figure 1 compares the age pyramid of the ENSANUT-2018 against the results of ENSANUT 2012. Differences greater than 2% are not observed. Furthermore, ENSANUT-2018 and ENSANUT 2012 practically coincided in the percentage of men in households: (48%).

Figure 1. Comparison of the age pyramids of the household population of ENSANUT 2012 (N=194,750) and ENSANUT 2018 (N=158,044)

| ENSANUT-2012 | ENSANUT 2018 |
| --- | --- |
|  |  |

1. Diabetes was determined by self-declaration or measurement of glucose/ glycosylated hemoglobin. Table 1 [1] compares estimators of the prevalence of diabetes (diagnosed+undiagnosed) on adults (20+ years old) of ENSANUT-2020 and ENSANUT 2018; no statistically significant differences are observed.

Table 2. Prevalence of diabetes (diagnosed+ undiagnosed) by age.

| Age group (years old) | ENSANUT 2020 | ENSANUT 2018 | ¿ Do intervals overlap? |
| --- | --- | --- | --- |
| 20-29 | 2.2% (1.3,1.8) | 3.2% (1.7,6.2) | Yes |
| 30-39 | 7.6% (5.4,10.7) | 7.7% (6.2,9.5) | Yes |
| 40-49 | 18.6% (14.3,23.8) | 16.2% (13.8,18.8) | Yes |
| 50-59 | 28.6% (23.0,35.0) | 28.9% (25.7,32.3) | Yes |
| 60-69 | 28.1% (22.3,34.8) | 34.0% (29.9,38.3) | Yes |
| 70+ | 29.5% (22.6,37.6) | 29.5% (25.5,33.7) | Yes |
| Total | 15.7% (13.9,17.6) | 16.8% (15.6,18.1) | Yes |

1. References

[1] Ana Basto-Abreu, Nancy López-Olmedo, Rosalba Rojas-Martínez, Carlos A. Aguilar-Salinas, Vanessa De la Cruz-Góngora, Juan A. Rivera, Teresa Shamah-Levy, Martin Romero-Martinez, Simón Barquera, Salvador Villalpando, Tonatiuh Barrientos-Gutierrez. Prevalence of diabetes and glycemic control in Mexico: national results from 2018 and 2020. Technical Report
